# Inflammatory cytokine oncostatin M induces endothelial activation in macro- and microvascular endothelial cells and in APOE*3Leiden.CETP mice

**DOI:** 10.1371/journal.pone.0204911

**Published:** 2018-10-01

**Authors:** Danielle van Keulen, Marianne G. Pouwer, Gerard Pasterkamp, Alain J. van Gool, Maarten D. Sollewijn Gelpke, Hans M. G. Princen, Dennie Tempel

**Affiliations:** 1 Laboratory of Experimental Cardiology, University Medical Centre Utrecht, Utrecht, The Netherlands; 2 Laboratory of Clinical Chemistry and Haematology, University Medical Centre Utrecht, Utrecht, The Netherlands; 3 Quorics B.V, Rotterdam, The Netherlands; 4 TNO-Metabolic Health Research, Gaubius Laboratory, Leiden, The Netherlands; 5 Department of Cardiology, Leiden University Medical Center, Leiden, The Netherlands; 6 TNO- Microbiology & Systems Biology, Zeist, The Netherlands; 7 Radboudumc, Nijmegen, The Netherlands; 8 Molecular Profiling Consulting, London, England; Max Delbruck Centrum fur Molekulare Medizin Berlin Buch, GERMANY

## Abstract

**Aims:**

Endothelial activation is involved in many chronic inflammatory diseases, such as atherosclerosis, and is often initiated by cytokines. Oncostatin M (OSM) is a relatively unknown cytokine that has been suggested to play a role in both endothelial activation and atherosclerosis. We comprehensively investigated the effect of OSM on endothelial cell activation from different vascular beds and in APOE*3Leiden.CETP mice.

**Methods and results:**

Human umbilical vein endothelial cells, human aortic endothelial cells and human microvascular endothelial cells cultured in the presence of OSM express elevated *MCP-1*, *IL-6* and *ICAM-1* mRNA levels. Human umbilical vein endothelial cells and human aortic endothelial cells additionally expressed increased VCAM-1 and E-selectin mRNA levels. Moreover, ICAM-1 membrane expression is increased as well as MCP-1, IL-6 and E-selectin protein release. A marked increase was observed in STAT1 and STAT3 phosphorylation indicating that the JAK/STAT pathway is involved in OSM signaling. OSM signals through the LIF receptor alfa (LIFR) and the OSM receptor (OSMR). siRNA knockdown of the LIFR and the OSMR revealed that simultaneous knockdown is necessary to significantly reduce MCP-1 and IL-6 secretion, VCAM-1 and E-selectin shedding and STAT1 and STAT3 phosphorylation after OSM stimulation. Moreover, OSM administration to APOE*3Leiden.CETP mice enhances plasma E-selectin levels and increases ICAM-1 expression and monocyte adhesion in the aortic root area. Furthermore, *Il-6* mRNA expression was elevated in the aorta of OSM treated mice.

**Conclusion:**

OSM induces endothelial activation *in vitro* in endothelial cells from different vascular beds through activation of the JAK/STAT cascade and *in vivo* in APOE*3Leiden.CETP mice. Since endothelial activation is an initial step in atherosclerosis development, OSM may play a role in the initiation of atherosclerotic lesion formation.

## 1. Introduction

The endothelium is involved in many processes including maintenance of the endothelial barrier function, prevention of spontaneous blood clot formation, inflammatory cell recruitment upon injury and regulation of the vascular tone[1–3]. Impairment of one or more of these functions is often referred to as endothelial dysfunction, and may lead to the development of atherosclerosis, angiogenesis in cancer, vascular leakage, infectious diseases or stroke[4].

Although endothelial dysfunction is often described as the inability to dilate vessels, endothelial dysfunction is also characterized by endothelial activation, which is marked by increased cytokine release, adhesion molecule expression and endothelial permeability. The released cytokines attract leukocytes to the site of the activated endothelium, where the leukocytes bind to the endothelial barrier, which is enabled by enhanced adhesion molecule expression. Firmly adhered leukocytes then migrate through the endothelial barrier into the underlying tissue[5].

The process of endothelial activation can occur both, locally on well-known predilection sites and systemically, and is often triggered by traditional cardiovascular risk factors such as hypercholesterolemia, hypertension, smoking or diabetes and is initiated by inflammatory cytokines. One such a cytokine, which was first discovered in the cancer field, is oncostatin M (OSM). This relatively unexplored cytokine is an interleukin-6 family member that can signal through the LIFR and the OSMR, which are both dependent on heterodimerization with the gp130 receptor to form a functional receptor complex[6]. OSM is upregulated in multiple chronic inflammatory diseases including periodontitis, rheumatoid arthritis and inflammatory bowel diseases and is known to induce angiogenesis and smooth muscle cell proliferation and migration, both processes that are involved in atherosclerosis development[7–16]. Other pro-inflammatory cytokines that promote angiogenesis, smooth muscle cell proliferation and endothelial activation, such as TNFα and IL-18, have already been proven to accelerate atherosclerosis[17–24]. Furthermore, OSM is found in human carotid atherosclerotic plaques and in the intima and media of atherosclerotic mice[16].

Based on these findings and on the knowledge that endothelial cells are very high expressers of OSM receptors[25], we hypothesized that OSM may be involved in atherosclerosis development partially by inducing endothelial activation as a first step in the development of atherosclerosis. In this study, we incubated human endothelial cells with OSM to investigate if OSM induces systemic or local endothelial activation. As the cell heterogeneity among endothelial cells is huge[26,27] and endothelial cells from different vascular beds show different responses/ behave different to physiological stimuli[28,29], we tested the effect of OSM in endothelial cells derived from multiple vascular beds, human umbilical vein endothelial cells (HUVECs), human aortic endothelial cells (HAECs) and human microvascular endothelial cells (HMEC-1). Of which HAECs are the most suitable endothelial cell type to study atherosclerosis development as atherosclerosis mainly affects the medium and large-sized arteries[30]. To validate our findings in cultured endothelial cells *in vivo*, we administered OSM to APOE*3Leiden.CETP mice, a translational mouse model for hyperlipidemia and atherosclerosis[31,32]. The mildly pro-inflammatory state that is present in this animal model of hyperlipidemia makes it a suitable model to investigate the role of OSM in atherosclerosis prone conditions. We found that OSM induces endothelial activation in all different investigated human endothelial cell types and in mice after chronic administration and identified the JAK/STAT pathway as a key player in this process.

## 2. Materials and methods

### 2.1 Cell culture

2 different batches of pooled primary human umbilical vein endothelial cells (HUVECs, Lonza, the Netherlands), a single batch of primary human aortic endothelial cells from one single donor (HAECs, ATCC, Manassas, VA, USA) and a human dermal microvascular endothelial cell line (HMEC-1, ATCC, Manassas, VA, USA) were cultured in EBM^®^-2 medium (Lonza, Walkersville, MD) supplemented with EGM^TM^-2 SingleQuots^®^ (Lonza, Walkersville, MD) under normoxic conditions (21% O_2_). Throughout the study, passage 6 was used for HUVECs and HAECs, while passage 27 was used for the HMEC-1 cell line. All experiments were performed in 70% subconfluent HUVECs, HAECs, or HMEC-1 cells. After each experiment, cells and conditioned medium were collected for subsequent RNA or protein analysis. Repetitive experiments were only started if the previous experiment had been finished.

### 2.2 *In vitro* RNA expression

Human OSM (R&D systems, Minneapolis, MN) was added to HUVECs, HAECs and HMEC-1 cells in a concentration range from 0–20 ng/mL. After 3 or 6 hours, RNA was isolated with the NucleoSpin^®^ RNA kit (Macherey-Nagel, Düren, Germany) according to the manufacturer’s protocol. Isolated RNA (500 ng) was reverse transcribed into cDNA with the qSCript™ cDNA Synthesis Kit (Quanta Biosciences, Beverly, MA) and analyzed by real-time fluorescence assessment of SYBR Green signal (iQ™ SYBR^®^ Green Supermix, Bio-Rad, Hercules, CA) in the CFX96™ Real-Time Detection System (Bio-Rad, Hercules, CA). Each sample was measured in duplicates. Primers were designed for the human genes of interest, sequences are listed in Table 1. MRNA levels were analyzed and corrected for the housekeeping gene *ACTB*. Experiments were repeated 4–7 times.

**Table 1:** Primer sets for qPCR analysis.

Gene	Species	Direction	Primer sequence (5’-3’)
*MCP-1*	Human	Forward	TGGAATCCTGAACCCACTTCT
		Reverse	CAGCCAGATGCAATCAATGCC
*IL-6*	Human	Forward	AGTGAGGAACAAGCCAGAGC
		Reverse	GTCAGGGGTGGTTATTGCAT
*ICAM-1*	Human	Forward	TTGAACCCCACAGTCACCTAT
		Reverse	CCTCTGGCTTCGTCAGAATCA
*VCAM-1*	Human	Forward	TGGGAAAAACAGAAAAGAGGTG
		Reverse	GTCTCCAATCTGAGCAGCAA
*E-SELECTIN*	Human	Forward	AAGCCTTGAATCAGACGGAA
		Reverse	TCCCTCTAGTTCCCCAGATG
*ACTB*	Human	Forward	GATCGGCGGCTCCATCCTG
		Reverse	GACTCGTCATACTCCTGCTTGC
*Mcp-1*	Murine	Forward	TTAAAAACCTGGATCGGAACCAA
		Reverse	GCATTAGCTTCAGATTTACGGGT
*Il-6*	Murine	Forward	CTATACCACTTCACAAGTCGGA
		Reverse	GAATTGCCATTGCACAACTCTTT
*Icam-1*	Murine	Forward	TCCGCTACCATCACCGTGTAT
		Reverse	TAGCCAGCACCGTGAATGTG
*Hprt*	Murine	Forward	TCAGGAGAGAAAGATGTGATTGA
		Reverse	CAGCCAACACTGCTGAAACA

### 2.3 *In vitro* cytokine release

To determine the effect of OSM on endothelial activation, HUVECs, HAECs or HMEC-1 cells were incubated with 5 ng/mL OSM. 3h and 6h after OSM treatment, conditioned medium was collected. To investigate the effect of OSM on endothelial activation after siRNA knockdown of the LIFR and OSMR, siRNA transfected HUVECs were treated with 5 ng/mL OSM 48h post transfection. 6h after OSM treatment conditioned medium was collected. Conditioned medium was analyzed with the ProcartaPlex Mix&Match Human 6-plex (Thermo Fisher, Waltham, MA) according to the manufacturer’s protocol and measured on the Bio-plex^®^ 200 system (Bio-Rad, Hercules, CA) to determine the release of MCP-1, IL-6, soluble E-selectin, soluble P-selectin and soluble VCAM-1. Experiments were repeated 3–7 times.

### 2.4 Flow cytometry

5 ng OSM was added to HUVECs, HAECs, or HMEC-1 cells for 18h. Cells were washed with PBS and detached with accutase. Subsequently, cells were fixed with 1% PFA and incubated with 2.5 μL antibodies/ 1,000,000 cells against VCAM-1, ICAM-1, P-selectin and, E-selectin all obtained from Thermo Fisher (S1 Table). The experiment was repeated 3 times.

### 2.5 siRNA transfection

Knockdown of LIFR and OSMR was achieved by transfection with a mix of 4 specific siRNA sequences directed against the human mRNA sequence (SMARTpool siGENOME, GE Dharmacon, Lafayette, CO) in 70% subconfluent HUVEC cultures. Cells were incubated for 1 hour in a small volume of EGM-2 medium supplemented with DharmaFECT 1 (GE Dharmacon, Lafayette, CO) according to manufacturer’s instructions. After 2 hours cells were supplemented with extra EGM-2 medium to complement medium volumes. As controls, HUVECs were transfected with a mix of 4 scrambled, non-targeting siRNAs (siSham Smartpool; GE Dharmacon, Lafayette, CO). siRNA transfected HUVECs were treated with OSM 48h after siRNA transfection.

### 2.6 Western blot

HUVECs were lysed with cOmplete™ Lysis-M, EDTA-free reagent (Sigma Aldrich, Saint Louis, MO) for 15 minutes on ice. Next, protein concentration was determined with the Pierce™ BCA protein Assay Kit (Thermo Scientific, Waltham, MA). The protein sample was treated with NuPAGE™ Sample Reducing Agent (Thermo Scientific, Waltham, MA) and NuPAGE™ LDS Sample Buffer (Thermo Scientific, Waltham, MA). Subsequently, the solution was boiled at 70°C for 10 minutes. Samples were loaded on a Bolt™ 4–12% Bis-Tris Plus gel (Thermo Scientific, Waltham, MA), run for 50 minutes at 160V and transferred to an iBlot^®^2 PVDF Stack (Thermo Scientific, Waltham, MA) with the iBlot^®^2 Gel Transfer Device (Thermo Scientific, Waltham, MA). Blots were incubated with the primary antibody overnight at 4°C (S1 Table). Subsequently, blots were incubated with the appropriate secondary antibody conjugated with horseradish peroxidase (HRP) for 1h at RT (S1 Table). Peroxidase labeled antibodies were detected with Chemiluminescent Peroxidase Substrate (Sigma, Saint Louis, MO).

### 2.7 Animals and treatments

Thirty-two female APOE*3Leiden.CETP transgenic mice (15–22 weeks of age) were used. The number of animals per group was calculated with Java Applets for Power and Sample Size [Computer software], from http://homepage.stat.uiowa.edu/~rlenth/Power/index.html using a one-way ANOVA with a probability of 0.05 and a Dunnett’s correction, a SD of 20%, a power of 80% and a minimal expected difference of 35%. Mice were housed under standard conditions with a 12h light-dark cycle and had free access to food and water. Body weight, food intake and clinical signs of behavior were monitored regularly during the study. Mice received a Western type diet (WTD) (a semi-synthetic diet containing 15 w/w% cacao butter and 0.15% dietary cholesterol, Altromin, Tiel, the Netherlands). At T = 0 weeks, after a run-in period of 3 weeks, mice were matched based on plasma total cholesterol levels, plasma triglyceride levels, body weight, and age in 4 groups of 8 mice. Two mice died during the diet intervention period, 1 in the 1μg/kg/day OSM group and 1 in the 10μg/kg/day OSM group. At T = 7 weeks, an ALZET^®^ Osmotic Pump Type 1004 (4-week release duration, Durect, Cupertino, CA) containing either 1, 3 or 10 μg/kg/day murine OSM (R&D systems, Minneapolis, MN) or PBS was placed subcutaneously in the flank. Doses were based on previous studies, which gave a single or double injection of 5–50 μg/kg OSM resulting in local increased permeability, edema, swelling, infiltration of immune cells, increased serum VEGF levels and increased angiopoetin 2 expression[33–36]. All solutions, also PBS of control group, contained 1% mouse serum to prevent OSM from sticking to plastics. Prior to surgery, mice received the analgesic Carprofen (5 mg/kg s.c.) and were anesthetized with isoflurane (induction 4%, maintenance 2%). At T = 10 weeks, mice were euthanized by gradual CO_2_ inhalation (6 L/min in a 20 Liter container). CO_2_ flow was maintained for a minimum of 1 minute after respiration ceased (as observed by lack of respiration and faded eye color). Death was confirmed by exsanguination (via heart puncture). Hearts were isolated for immunohistochemistry in the aortic root and aortas were isolated for RNA expression analysis. EDTA blood samples were drawn after a 4 hour fast at T = 0 and T = 10 weeks. All animal experiments were performed conform the guidelines from Directive 2010/63/EU of the European Parliament on the protection of animals used for scientific purposes or the NIH guidelines. The care and use of all mice in this study was carried out at the animal facility of The Netherlands Organization for Applied Research (TNO) in accordance with the ethical review committee “TNO-DEC” under the registration number 3683. Animal experiments were approved by the Institutional Animal Care and Use Committee of TNO under registration number TNO-202.

### 2.8 Plasma parameters

Plasma cholesterol and triglycerides were measured spectrophotometrically with enzymatic assays (Roche diagnostics). The inflammatory markers, E-selectin and MCP-1 were measured with ELISA kits from R&D. Plasma ALT and AST were determined using a spectrophotometric assay (Boehringer Reflotron system) in group wise-pooled samples from sacrifice plasma. All assays were performed according to the manufacturer’s instruction.

### 2.9 Histological assessment of vascular inflammation

Vascular inflammation was assessed in the aortic root area as reported previously by Landlinger *et al*[37] in control mice and mice receiving 10 μg/kg/day OSM. Briefly, the aortic root was identified by the appearance of aortic valve leaflets and serial cross-sections of the entire aortic root area (5 μm thick with intervals of 50 μm) were mounted on 3-aminopropyl triethoxysilane-coated slides and stained with hematoxylin-phloxine-saffron (HPS). Each section consisted of 3 segments (separated by the valves) and in 4 sections ICAM-1 expression and the number of monocytes adhering to the activated endothelium was counted after immunostaining with mouse monoclonal ICAM-1 antibody (Santa Cruz) and AIA 31240 antibody (Accurate Chemical and Scientific) respectively (S1 Table). One mouse from the control group was excluded from analysis due to a technical error, resulting in 7 and 8 mice per group.

### 2.10 RNA isolation murine tissue

To isolate RNA from aortic tissue, RA1 lysis buffer (Macherey-Nagel, Düren, Germany) containing 1% DTT was added to the tissue, which was cut in tiny pieces and subsequently minced. RNA was isolated with the RNeasy^®^ Plus Micro Kit (Qiagen, Hilden, Germany) according to the RNeasy Fibrous Tissue Mini Kit protocol (Qiagen, Hilden, Germany). Isolated RNA (500 ng) was reverse transcribed into cDNA with the qSCript™ cDNA Synthesis Kit (Quanta Biosciences, Beverly, MA) and analyzed by real-time fluorescence assessment of SYBR Green signal (iQ™ SYBR^®^ Green Supermix, Bio-Rad, Hercules, CA) in the CFX96™ Real-Time Detection System (Bio-Rad, Hercules, CA). Each sample was measured in duplicates. Primers were designed for the murine genes of interest, sequences are listed in Table 1. mRNA levels were analyzed and corrected for the housekeeping gene *Hprt*. RNA isolation was unsuccessful in one mouse from the 3μg/kg/day OSM group resulting in 6, 7 and 8 mice per group.

### 2.11 Statistical analysis

qPCR data was analyzed according to the ΔΔCt method, statistical tests were performed on ΔCt values. Two-way-anova was used to analyze *in vitro* data to take into account day-to-day variation of the experiments. Not normally (Gaussian) distributed parameters were transformed with the natural logarithm or in case of undetectable values analyzed with the appropriate non-parametric test. Dose-dependency was determined by a Pearson correlation. All statistical analyses were performed in SPSS statistics version 21.0. A two-tailed p-value of 0.05 was regarded statistically significant in all analyses. Graphs were made in GraphPad Prism version 7.02 for Windows, GraphPad Software, La Jolla California USA, www.graphpad.com

## 3. Results

### 3.1 OSM induces endothelial activation in human endothelial cells

**Fig 1:**
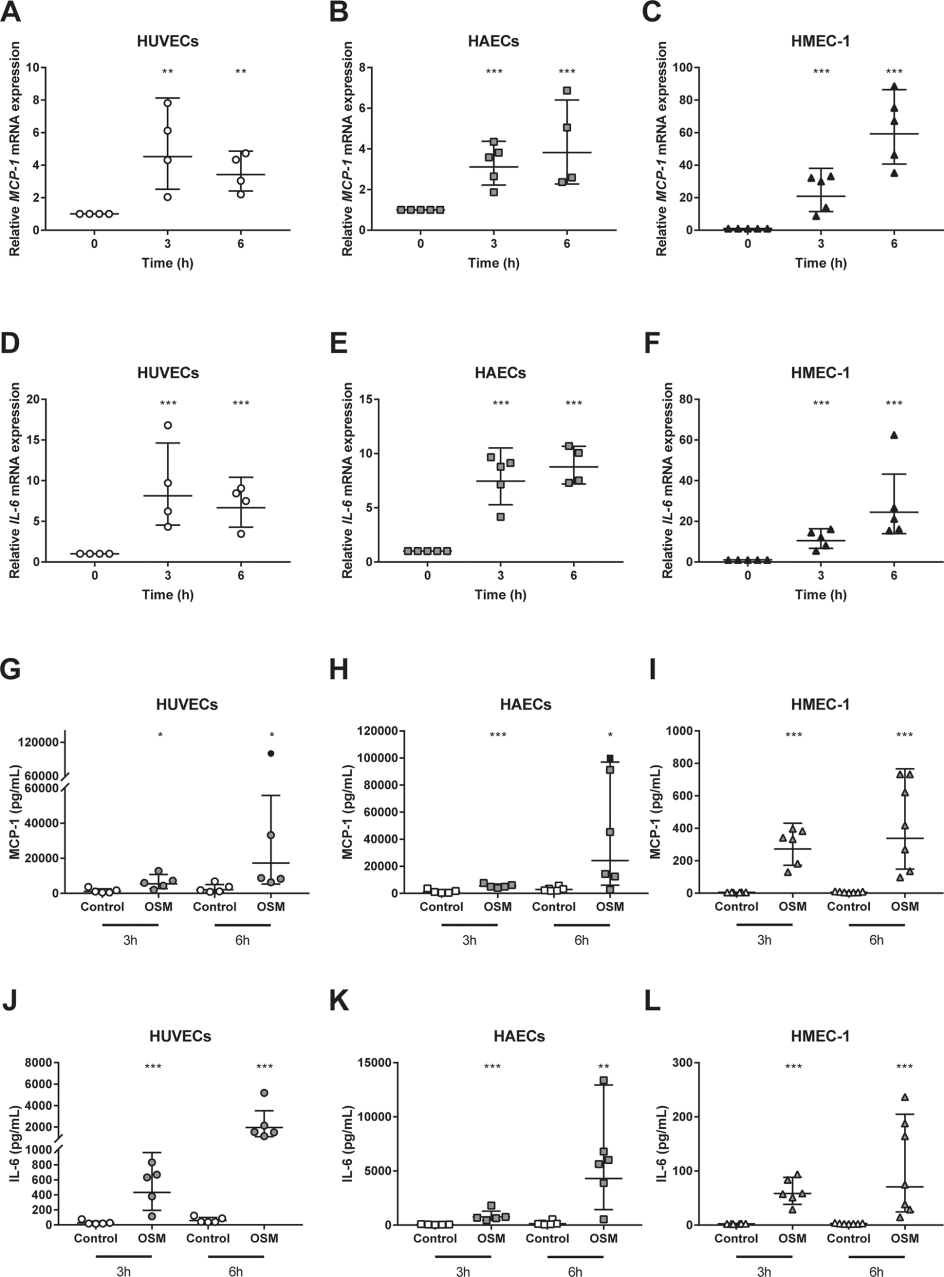
OSM increases cytokine release in different endothelial cells. HUVECs, HAECs and HMEC-1 cells were incubated with 5 ng/mL OSM for the indicated period of time. All values are relative values compared to control, which was given an arbitrary value of 1. Values were normalized to *ACTB* and calculated with the ΔΔCt method. A two-way ANOVA with Dunnett’s test was performed on the ΔCt values to test for significance (A-F). MCP-1 and IL-6 release was measured in conditioned medium of HUVECs, HAECs and HMEC-1 cells incubated with 5 ng/mL OSM for 3 or 6h. Values too high to measure were arbitrarily set on 100,000 and are indicated with ● or ■. Data sets without missing values were ln transformed and analyzed with an independent t-test for significance while data sets with missing values were analyzed with a Mann Whitney U test (G-L). All data represent geometric mean ± geometric SD. *p<0.05 **p<0.01 ***p<0.001 compared to control (n = 4–7).

**Fig 2:**
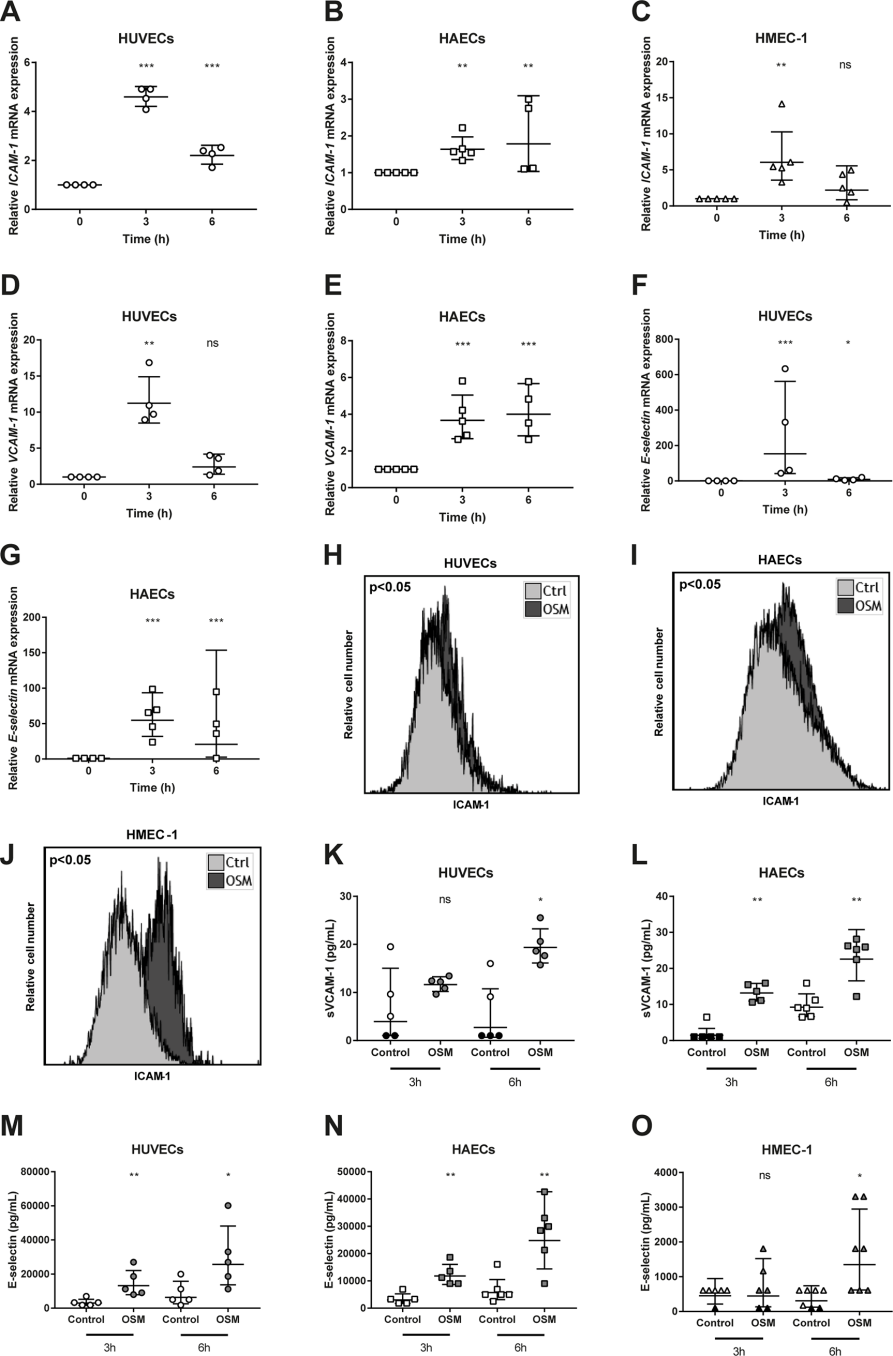
OSM increases adhesion molecule expression and release in different endothelial cells. HUVECs, HAECs and HMEC-1 cells were incubated with 5 ng/mL OSM for the indicated period of time. All values are relative values compared to control, which was given an arbitrary value of 1. Values were normalized to *ACTB* and calculated with the ΔΔCt method. A two-way ANOVA with Dunnett’s test was performed on the ΔCt values to test for significance (A-C). ICAM-1 membrane expression was determined in HUVECs, HAECs and HMEC-1 cells treated with 5 ng/mL OSM for 18h. A two-way ANOVA was used to test for significance (D-F). Shedding of VCAM-1 and E-selectin was determined in conditioned medium of HUVECs, HAECs and HMEC-1 cells treated with 5 ng/mL OSM for 3 or 6h by measuring soluble VCAM-1 and E-selectin. Soluble VCAM-1 values too low to measure were arbitrarily set on 1 and are indicated with ● or ■. Soluble E-selectin values too low to measure were arbitrarily set on 100 and are indicated with ▲. Data sets without missing values were ln transformed and analyzed with an independent t-test for significance while data sets with missing values were analyzed with a Mann Whitney U test (G-K). All data represent geometric mean ± geometric SD, except for flow cytometry data which shows a representative histogram of control and OSM treated cells (n = 3–7). *p<0.05 **p<0.01 ***p<0.001 compared to control, ns = not significant.

To investigate whether OSM induces endothelial activation, we first examined cytokine mRNA expression in HUVECs, HAECs and HMEC-1 cells treated with 5 ng/mL OSM for 3 or 6 hours. OSM treatment was found to increase mRNA expression of the cytokines *MCP-1* (p<0.01) and *IL-6* (p<0.001) in HUVECs, HAECs (p<0.001) and HMEC-1 cells (p<0.001) at both 3h and 6h time points (Fig 1A–1F). Since these cytokines are released by activated endothelial cells, we next measured MCP-1 and IL-6 protein concentrations in conditioned medium of OSM treated HUVECs, HAECs and HMEC-1 cells. Both MCP-1 (p<0.05) and IL-6 (<0.001) release were increased in OSM treated HUVECs, HAECs (p<0.05 and p<0.01 respectively) and HMEC-1 cells (p<0.001) at both time points (Fig 1G–1L). Subsequently, we measured adhesion molecule expression, which is another feature of endothelial activation. *ICAM-1* mRNA expression was increased by OSM treatment in HUVECs (p<0.001) and HAECs (p<0.01) again at both 3h and 6h time points and in HMEC-1 cells 3h after addition of OSM (p<0.01)(Fig 2A–2C). *VCAM-1* mRNA expression was upregulated in HUVECs at 3h (p = 0.008) and in HAECs at both 3h and 6h (p<0.001)(Fig 2D and 2E). Moreover, we observed an upregulation in *E-selectin* mRNA expression in both HUVECs and HAECs at both 3h and 6h (p<0.001 and p<0.05)(Fig 2F and 2G), while *VCAM-1* and *E-selectin* mRNA levels were too low expressed in HMEC-1 cells. In addition, ICAM-1 membrane expression was increased in HUVECs (p<0.05), HAECs (p<0.05) and HMEC-1 cells (p<0.05) (Fig 2H–2J), but not membrane expression of VCAM-1, P-selectin or E-selectin (S1 Fig). Since these adhesion molecules can also be shed upon endothelial activation[38], we measured P-selectin, E-selectin, soluble VCAM-1 and soluble ICAM-1 levels in conditioned medium. Soluble VCAM-1 was upregulated in conditioned medium of HUVECs 6h after OSM addition (p<0.05) and in HAECs at both 3h and 6h post OSM addition (p<0.01) (Fig 2K and 2L). Soluble VCAM-1 was not detectable in conditioned medium of HMEC-1 cells. Additionally, E-selectin levels were upregulated at both time points in conditioned medium of OSM treated HUVECs (p<0.05) and HAECs (p<0.01) and 6h post OSM addition in HMEC-1 cells (p<0.05) (Fig 2M–2O). P-selectin levels were not detectable. Overall, these results indicate that OSM consistently induces endothelial activation *in vitro* in the different human endothelial cell types. Therefore, subsequent mechanistic studies were conducted in HUVECs.

## 3.2 JAK/STAT signaling is involved in OSM induced endothelial activation

**Fig 3:**
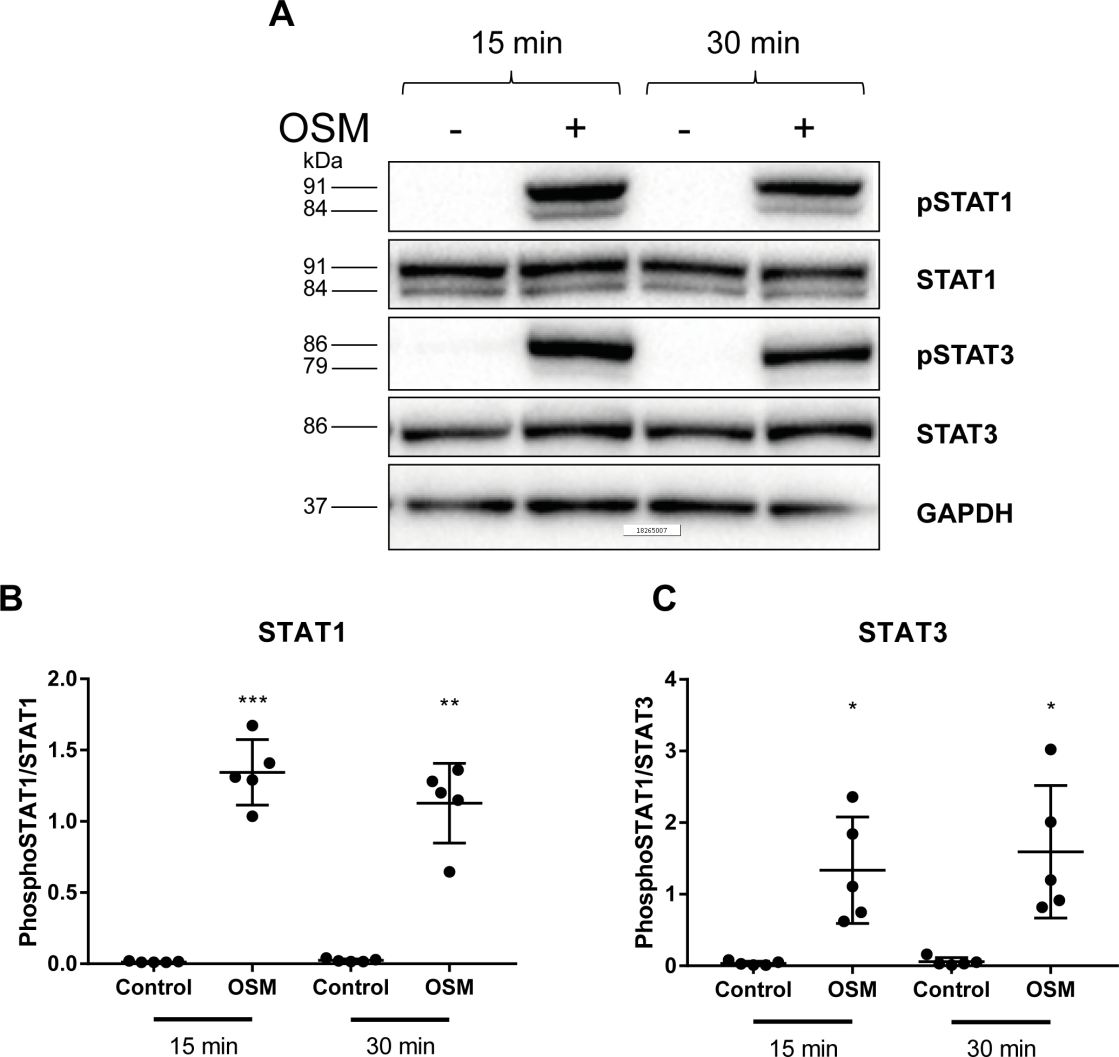
JAK/STAT pathway is involved in OSM induced endothelial activation. HUVECs were incubated with 5 ng/mL OSM for 15 or 30 min. A representative picture shows STAT1 phosphorylation at Tyr701, STAT1, STAT3 phosphorylation at Tyr705, STAT3 and GAPDH (A). Bar graphs show relative STAT1 and STAT3 phosphorylation (B,C). A two-way ANOVA was performed to test for significance. Data represent mean ± SD (n = 5). *p<0.05 **p<0.01 ***p<0.001 compared to control.

IL-6 family members signal through the Janus kinase/signal transducers and activators of transcription (JAK/STAT) pathway, a pathway that is often involved in cytokine and growth factor signaling[39–41]. Therefore, we investigated whether this pathway is also involved in OSM induced endothelial activation. STAT1 and STAT3 phosphorylation were markedly increased (p<0.05) (Fig 3) upon addition of OSM indicating that the JAK/STAT pathway is involved in OSM induced endothelial activation as well.

### 3.3 OSM induces endothelial activation by simultaneous signaling through the LIFR and OSMR

**Fig 4:**
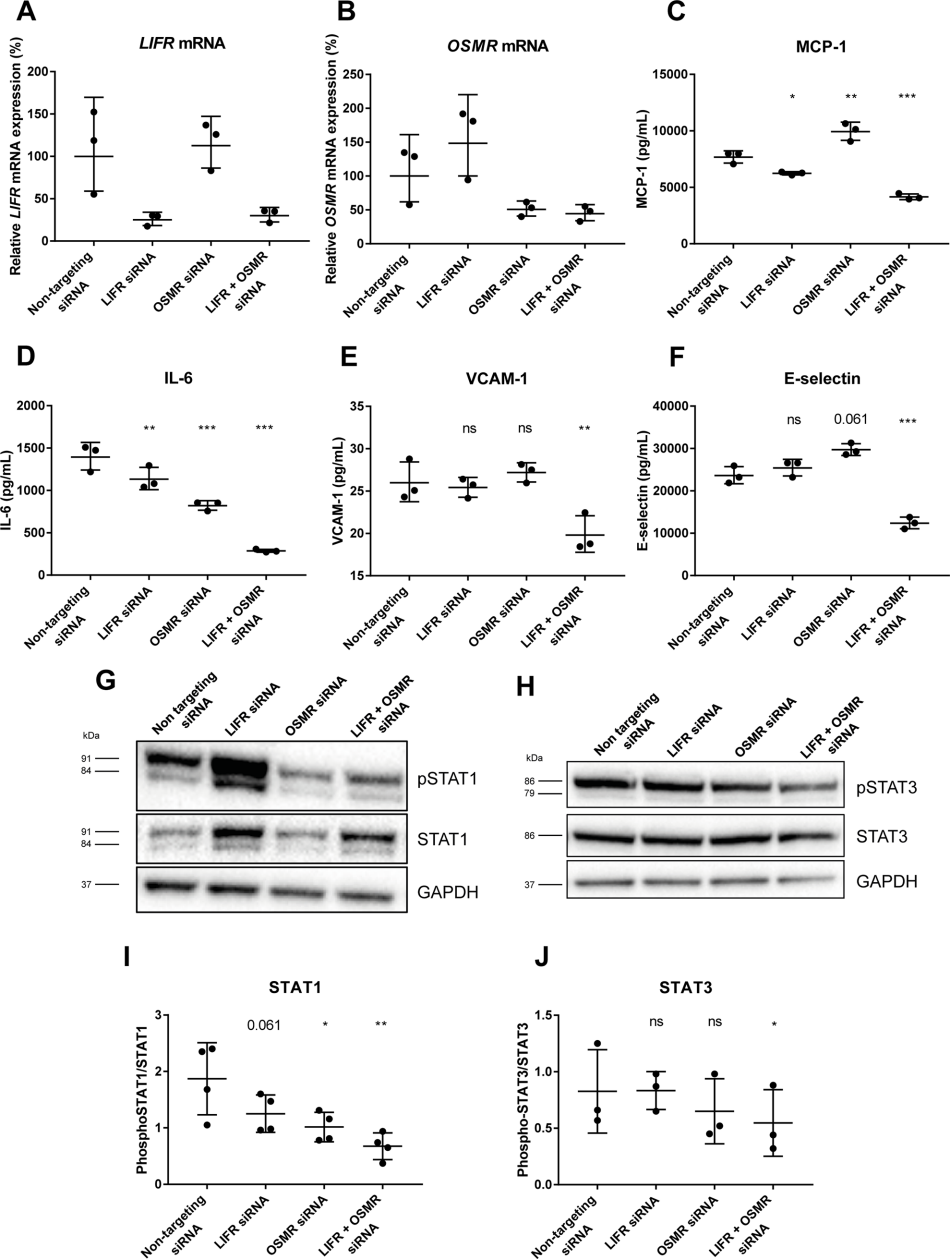
Simultaneous downregulation of LIFR and OSMR decreases IL-6 and MCP-1 release and prevents STAT1 and STAT3 phosphorylation. *LIFR* (A) and *OSMR* (B) mRNA expression levels were downregulated by siRNA transfection in HUVECs. 48h post transfection, HUVECs were treated with 5 ng/mL OSM for 6h to determine IL-6 and MCP-1 secretion and VCAM-1 and E-selectin shedding (C-F) or for 15 min to determine STAT1 and STAT3 phosphorylation (G-J). A two-way ANOVA with Dunnett’s test was performed to test for significance. All data represent mean ± SD (n = 3–4). *p<0.05 **p<0.01 compared to control, ns = not significant.

As OSM can signal through both the OSMR and the LIFR, a siRNA knockdown was performed to investigate which of these receptors is involved in OSM induced endothelial activation. *LIFR* mRNA expression was decreased to 25 ± 6% (mean ± SD), and *OSMR* mRNA expression to 52 ± 15%. Simultaneous knockdown resulted in a decrease of the *LIFR* to 31 ± 8% and of the *OSMR* to 45 ± 11% (Fig 4A and 4B). Single knockdown of LIFR did significantly decrease MCP-1 (p = 0.019) and IL-6 secretion (p = 0.005), but not VCAM-1 or E-selectin shedding. Single knockdown of OSMR did only decrease IL-6 secretion (p<0.001), while MCP-1 secretion was significantly increased (p = 0.007). VCAM-1 and E-selectin shedding were both not significantly changed. Double knockdown did not only decrease IL-6 (p<0.001) and MCP-1 (p<0.001) secretion, but also VCAM-1 (p = 0.009) and E-selectin (p<0.001) shedding compared to non-targeting siRNA treated cells (Fig 4C and 4D). A similar effect was observed for STAT1 and STAT3 phosphorylation, which was only reduced by double knockdown (p<0.05) compared to control (Fig 4E and 4F). Altogether, these data indicate that OSM signals through LIFR and OSMR simultaneously in human endothelial cells.

### 3.4 OSM induces an inflammatory response in APOE*3Leiden.CETP mice

**Fig 5:**
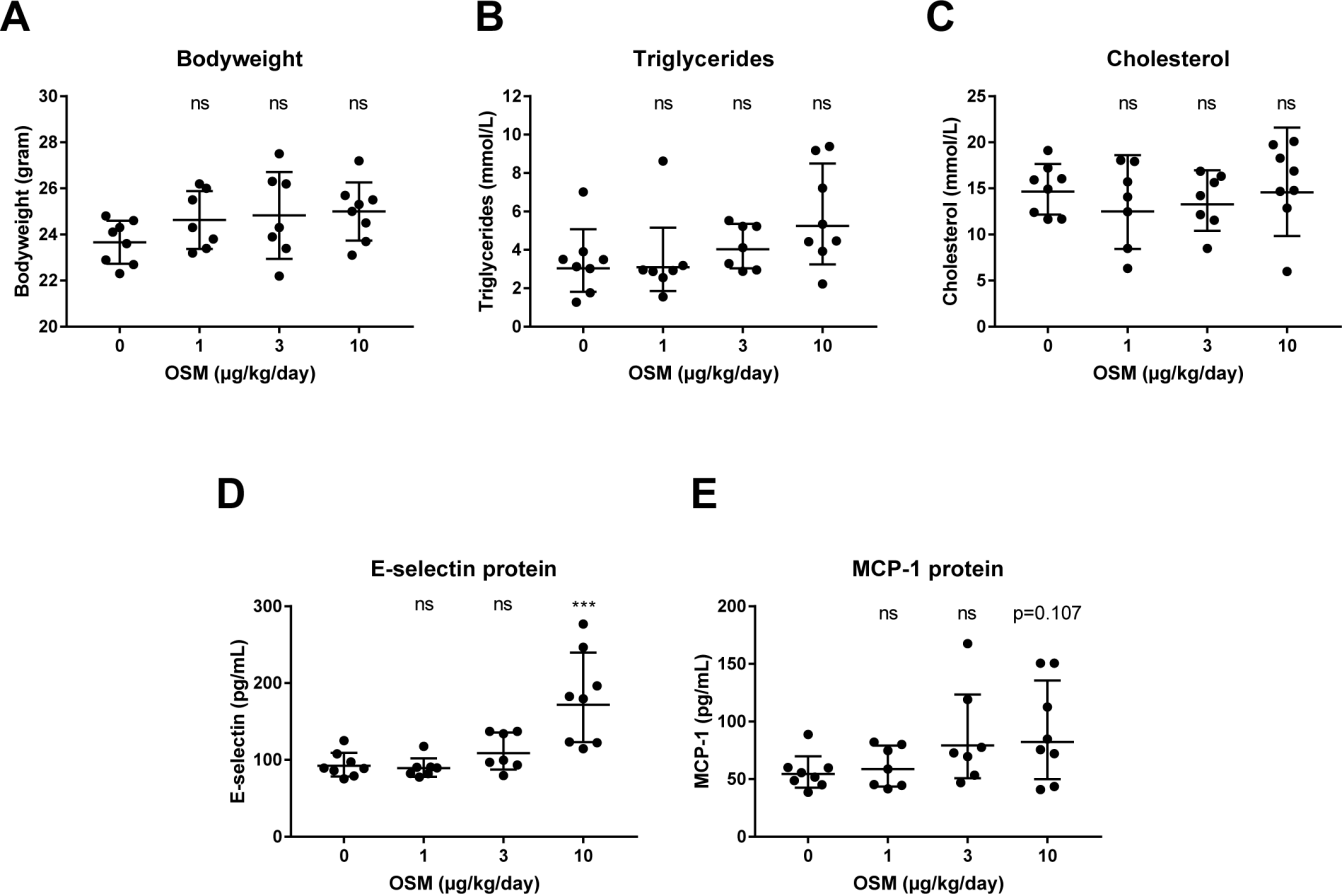
OSM enhances plasma levels of inflammatory markers in APOE*3Leiden.CETP mice treated with OSM. After 3 weeks of OSM treatment, bodyweight (A), triglyceride (B), cholesterol (C), E-selectin (D) and MCP-1 (E) levels were measured and compared to control mice. A one-way ANOVA with Dunnett’s test was performed on ln transformed data, except for the bodyweight, to test for significance. All data represent geometric mean ± geometric SD, except for bodyweight which represents mean ± SD (n = 7–8). ***p<0.001 compared to control, ns = not significant.

To investigate whether OSM activates the endothelium *in vivo* as well, hyperlipidemic APOE*3Leiden.CETP mice were administered OSM for 3 weeks. No clinical signs of deviant behavior and no significant effects on food intake were noted in any treatment group as compared to control. Plasma ALT and AST, measured at end-point as safety markers, showed no aberrant results (S2 Table). Also, no significant difference in body weight, triglyceride, or cholesterol levels were observed compared to control (Fig 5A–5C). As endothelial activation goes hand in hand with a pro-inflammatory response, plasma levels of inflammatory markers MCP-1 and E-selectin were measured. Plasma MCP-1 tended to be increased (p = 0.107) and plasma E-selectin was increased (p<0.001) in mice treated with 10 μg/kg/day OSM compared to the control group (Fig 5D and 5E).

### 3.5 OSM induces endothelial activation in the vasculature of APOE*3Leiden.CETP mice

**Fig 6:**
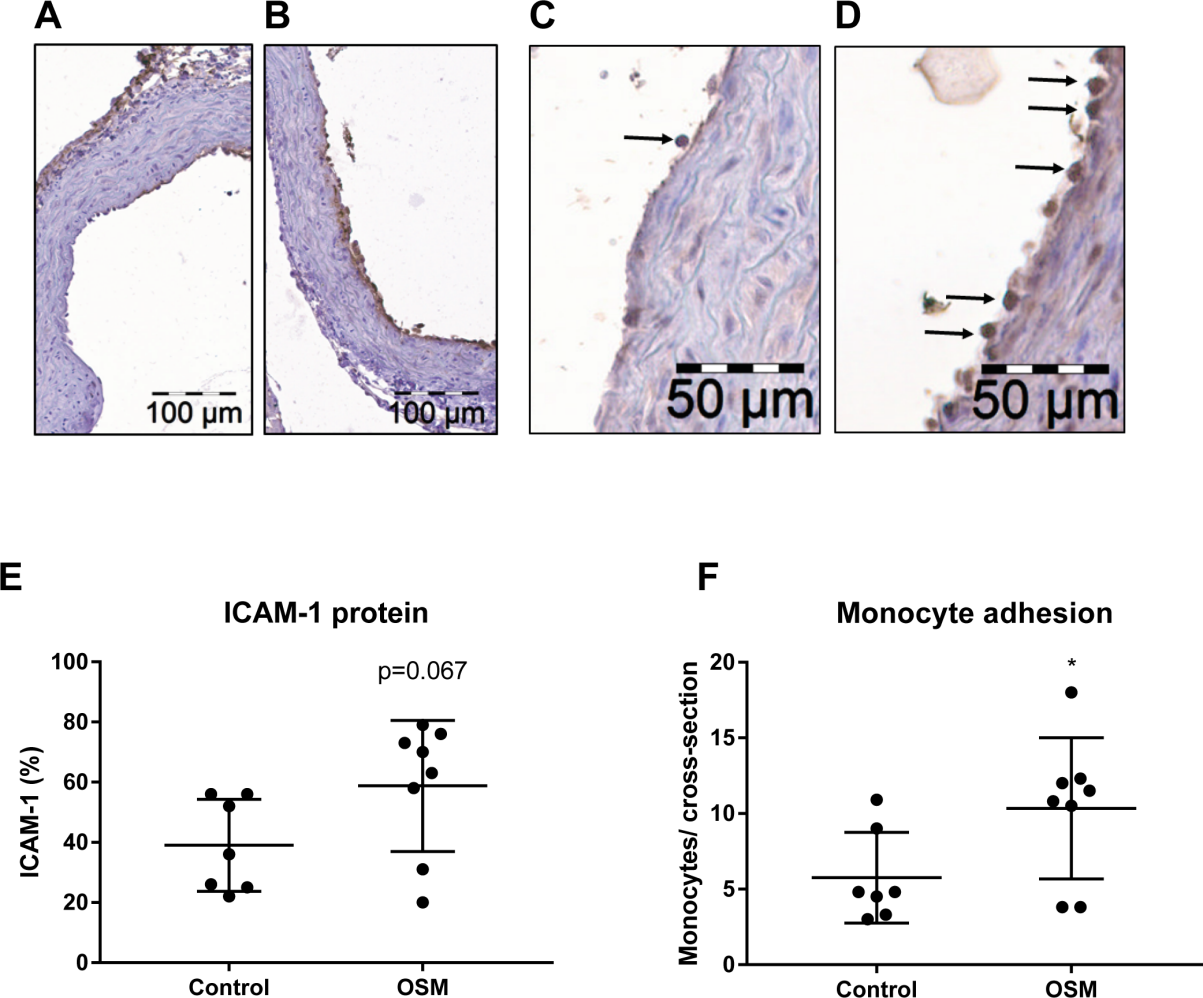
OSM increases ICAM-1 expression and monocyte adherence in the aortic root area in OSM treated APOE*3Leiden.CETP. Representative pictures showing the endothelial ICAM-1 expression (brown staining) in a control (A) and a 10 μg/kg/day OSM treated (B) mouse and monocyte adherence (arrows) in a control (C) and a 10 μg/kg/day OSM treated (D) mouse. Endothelial ICAM-1 expression was determined as percentage of the endothelial surface in the cross sections (E) and adhering monocytes were counted per cross-section after staining with AIA 31240 (F). Data represent mean ± SD (n = 7–8). An independent t-test was used to test for significance. Data represent mean ± SD. *p<0.05.

**Fig 7:**
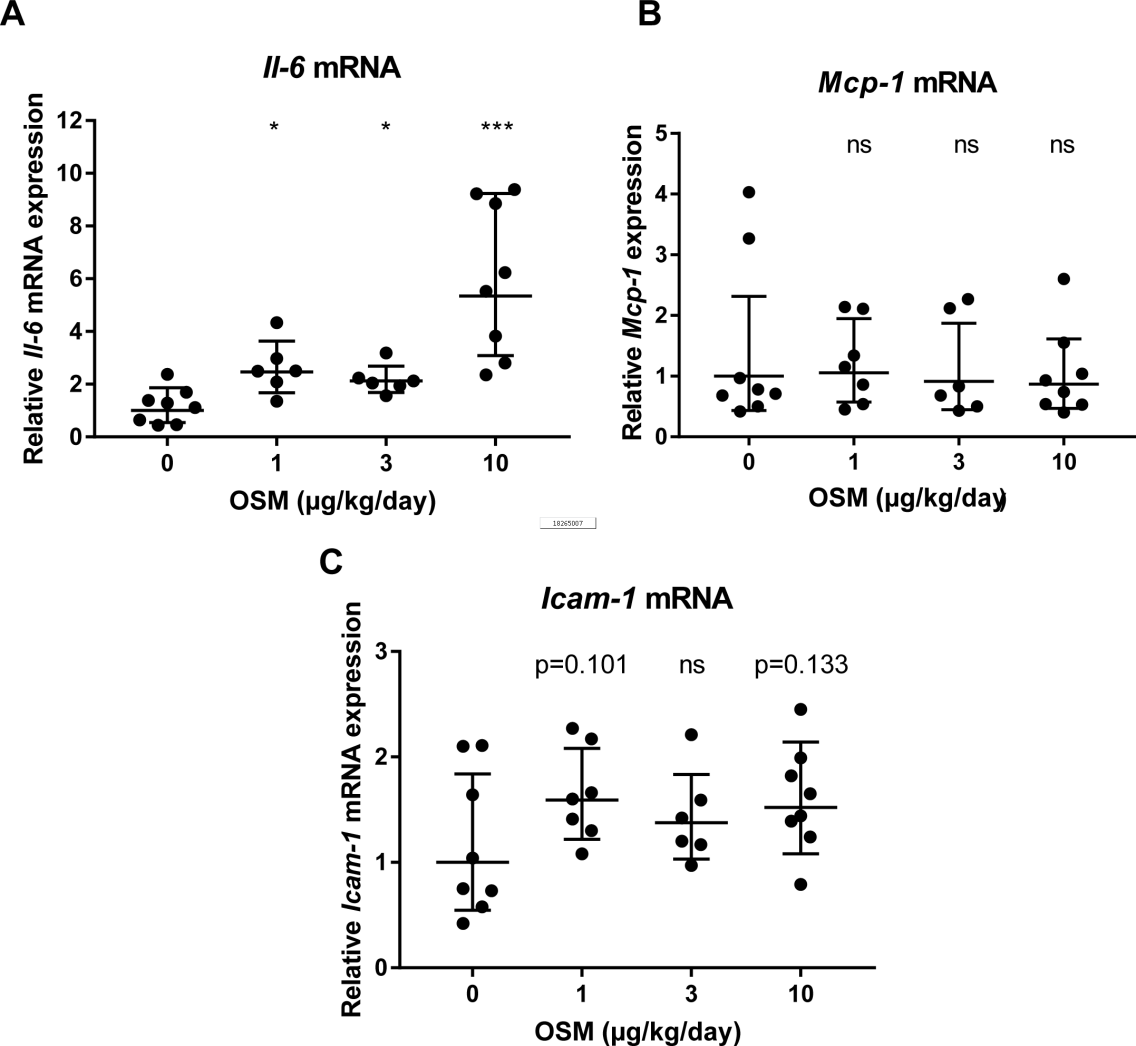
OSM increases *Il-6* mRNA expression in aortic tissue of APOE*3Leiden.CETP mice treated with OSM. After 3 weeks of OSM treatment, mRNA was isolated from the aorta and analyzed by qPCR. *Il-6* (A), *Mcp-1* (B) and *Icam-1* (C) mRNA expression were quantified. All values are relative values compared to the control group, which was given an arbitrary value of 1. Values were normalized to HPRT and calculated with the ΔΔCt method. A one-way ANOVA with Dunnett’s test was performed on the ΔCt values to test for significance. Data represent geometric mean ± geometric SD (n = 6–8). All values were compared to control. *p<0.05 ***p<0.001 compared to control, ns = not significant.

To further investigate if OSM is able to induce endothelial activation, the aortic root area was examined for relevant markers. ICAM-1 protein expression tended to be elevated from 39 ± 15% (mean ± SD) to 59 ± 22% (p = 0.067) and an increase in monocyte adhesion to the activated endothelium was observed from 5.7 ± 3.0 to 10.3 ± 4.7 monocytes (mean ± SD, p<0.05) in mice treated with 10μg/kg/day OSM (Fig 6). Furthermore, aortic mRNA expression analysis revealed a dose-dependent increase in *Il-6* expression (p<0.001) and *Icam-1* expression tended to be increased in the 1μg/kg/day and 10μg/kg/day OSM treated groups (p = 0.101 and p = 0.133 respectively) compared to control. *Mcp-1* mRNA expression was not enhanced (Fig 7). These results show that OSM does not only induce endothelial activation *in vitro*, but also *in vivo* in a hyperlipidemic mouse model.

## 4. Discussion

The present study demonstrates that OSM induces endothelial activation in cultured human endothelial cells as well as *in vivo* in APOE*3Leiden.CETP mice. The data show increased release of inflammatory markers and adhesion molecule expression, both features of endothelial activation. Furthermore, OSM increased monocyte adhesion in the aortic root area, as functional marker of endothelial activation.

We studied OSM induced endothelial activation *in vitro* by investigating the effect of OSM in three different types of human endothelial cells. Our data add to and expand on previous data that showed that OSM increases IL-6, IL-8 and MCP-1 secretion, ICAM-1 and VCAM-1 membrane expression and PMN adhesion to endothelial cells *in vitro* [34,42,43]. Consistently, increased VCAM-1 and E-selectin shedding was observed in all three endothelial cell types. ICAM-1 is an important adhesion molecule in monocyte binding as ICAM-1^-/-^ endothelial cells show a strong attenuation in monocyte binding compared to control endothelial cells[44]. Although we did not observe an increase in membrane E-selectin and VCAM-1 expression, OSM did increase soluble E-selectin and VCAM-1. Soluble VCAM-1 was previously shown to serve as a monocyte chemoattractant agent and soluble E-selectin enhances leukocyte migration and binding to endothelial cells[45,46]. Taken together, these observations show that OSM induces different biomarkers of endothelial activation in cultured endothelial cells.

Previous short term *in vivo* studies in healthy wildtype mice with OSM administered for only 6 to 24 hours have shown signs of acute endothelial activation, such as increased angiopoetin 2 expression in cardiac tissue, increased plasma VEGF levels and increased permeability and infiltration of inflammatory cells[33–36]. It is important to note that publicly available datasets show that *Osmr* and *Lifr* mRNA are expressed in aortic endothelial cells from mice as well (data accessible at NCBI GEO database [47], accession GSE114805 and [48], accession GSE115618).

The aim of the present study was to investigate the effect of chronic OSM exposure on endothelial activation in a hyperlipidemic mouse model, the APOE*3Leiden.CETP mouse. This mouse model features elevated lipid levels, representing humans with hyperlipidemia and mild chronic inflammation who have an increased risk of developing atherosclerosis[31,32,37]. We found that OSM tended to increase plasma MCP-1 and significantly increased plasma E-selectin, both markers of activated or dysfunctional endothelium[5,49] after 3 weeks of chronic OSM administration. Moreover, mRNA expression of *Il-6* was increased dose-dependently in aortic tissue of OSM treated mice. We also observed a trend towards increased ICAM-1 expression in the aortic root of OSM treated mice and a markedly enhanced monocyte binding as functional marker of activated endothelium, thus demonstrating augmented endothelial activation. ICAM-1 expression and adhesion of monocytes are strongly related, as previous studies show increased monocyte binding upon enhanced ICAM-1 expression and decreased monocyte binding upon reduced ICAM-1 expression[44,50]. Collectively, these findings provide evidence that OSM does not only induce endothelial activation *in vitro*, but also *in vivo* on top of the inflammatory state that is present in hyperlipidemic mice, resulting in increased monocyte recruitment and adherence.

Even though, endothelial cells are directly activated by OSM *in vitro*, it is important to note that the *in vivo* situation is much more complex and other cell types may have contributed to the observed effects as well. For instance, the increase in plasma MCP-1 and cardiac *Il-6* expression can partly be caused by fibroblasts or smooth muscle cells, as these two cell types also show increased IL-6 and MCP-1 expression upon OSM treatment *in vitro*[51,52]. Furthermore, OSM can promote growth factor and cytokine release in cell types other than endothelial cells, these released growth factors and cytokines can in turn activate the endothelium, thereby inducing indirect endothelial activation[51–53]. An example of such a growth factor is vascular endothelial growth factor (VEGF), which can be upregulated by OSM in multiple cell types[35,54–56] and is known to induce endothelial activation by increasing adhesion molecule expression and leukocyte adhesion[57].

Although our *in vivo* study was not aimed at and was too short to investigate whether chronic OSM exposure aggravates atherosclerosis, our results do give clues that OSM may be involved in the initiation of the atherosclerotic process. Some of the diverse hallmarks of endothelial activation that we observed, have previously been associated with atherosclerosis development in humans[49,58]. Further indications come from reports showing that OSM is present in both murine and human plaques[16], and higher mRNA expression levels of OSM in PBMCs derived from coronary artery disease patients compared to healthy individuals[59]. Moreover, a recent paper showed that prevention of OSM signaling, as opposed to stimulation of OSM signaling in our study, in OSMR-β^-/-^ApoE^-/-^ mice resulted in less and smaller atherosclerotic lesions and less macrophages compared to ApoE^-/-^ mice[60].

Other studies have shown that partial inhibition of endothelial activation by knockdown of E-selectin, P-selectin, ICAM-1 or MCP-1 attenuates atherosclerosis development in mice[61,62]. Therefore, lowering of plasma OSM levels or intervention in OSM signaling might be worth investigating as a possible future approach in the treatment of atherosclerosis.

As it is currently unknown which of the OSM receptors is involved in OSM induced endothelial activation, we performed a siRNA knockdown of the LIFR and the OSMR. Single knockdown experiments showed that solely LIFR or OSMR downregulation is not sufficient to prevent OSM induced endothelial activation or JAK/STAT signaling. Only simultaneous knockdown of both receptors was able to dramatically decrease IL-6 and MCP-1 release, VCAM-1 and E-selectin shedding and STAT1 and STAT3 phosphorylation. Hence, it is essential to block both receptors simultaneously or to target OSM when considering intervening in OSM signaling as a possible future therapy. Targeting both receptors or OSM itself could be a relative safe approach since OSM^-/-^ mice are viable and healthy[63].

Taken together, our comprehensive study provides new evidence that OSM induces activation of human endothelial cells from different vascular beds and in APOE*3Leiden.CETP mice chronically treated with OSM. Moreover, we provided data indicating both receptors for OSM as well as OSM itself as potential therapeutic targets in atherosclerosis and other chronic inflammatory diseases in which endothelial activation is involved such as rheumatoid arthritis, abnormal angiogenesis and thrombosis[64–67].

## Supporting information

S1 Fig OSM does not affect membrane expression of VCAM-1, E-selectin and P-selectin in endothelial cells. HUVECs, HAECs and HMEC-1 cells were incubated with 5 ng/mL OSM for 18h. A two-way ANOVA with Dunnett’s test was performed on the median to test for significance (n = 3). ns = not significant.
(TIF)

S1 Table List of antibodies used for flow cytometry, immunohistochemistry and western blot. (PDF)

S2 Table Average food intake and ALT and AST levels in mice. (PDF)

## References

[1] Sandoo A, van Zanten JJCSV, Metsios GS, Carroll D, Kitas GD. The endothelium and its role in regulating vascular tone. Open Cardiovasc Med J. Bentham Science Publishers; 2010;4: 302–12. 10.2174/1874192401004010302 21339899 PMC3040999

[2] Rajendran P, Rengarajan T, Thangavel J, Nishigaki Y, Sakthisekaran D, Sethi G, et al The vascular endothelium and human diseases. Int J Biol Sci. Ivyspring International Publisher; 2013;9: 1057–69. 10.7150/ijbs.7502 24250251 PMC3831119

[3] Wu KK, Thiagarajan P. Role of endothelium in thrombosis and hemostasis. Annu Rev Med. 1996;47: 315–331. 10.1146/annurev.med.47.1.315 8712785

[4] Hadi HAR, Carr CS, Al Suwaidi J. Endothelial dysfunction: cardiovascular risk factors, therapy, and outcome. Vasc Health Risk Manag. Dove Press; 2005;1: 183–98. Available: http://www.ncbi.nlm.nih.gov/pubmed/17319104 17319104 PMC1993955

[5] Szmitko PE, Wang C-H, Weisel RD, de Almeida JR, Anderson TJ, Verma S. New Markers of Inflammation and Endothelial Cell Activation: Part 1. Circulation. 2003;108: 1917–1923. Available: http://circ.ahajournals.org/content/108/16/1917 10.1161/01.CIR.0000089190.95415.9F 14568885

[6] Mosley B, De Imus C, Friend D, Boiani N, Thoma B, Park LS, et al Dual oncostatin M (OSM) receptors. Cloning and characterization of an alternative signaling subunit conferring OSM-specific receptor activation. J Biol Chem. 1996;271: 32635–32643. 10.1074/jbc.271.51.32635 8999038

[7] O’Kane CM, Elkington PT, Friedland JS. Monocyte-dependent oncostatin M and TNF-α synergize to stimulate unopposed matrix metalloproteinase-1/3 secretion from human lung fibroblasts in tuberculosis. Eur J Immunol. WILEY‐VCH Verlag; 2008;38: 1321–1330. 10.1002/eji.200737855 18398932

[8] Brown TJ, Lioubin MN, Marquardt H. Purification and characterization of cytostatic lymphokines produced by activated human T lymphocytes. Synergistic antiproliferative activity of transforming growth factor beta 1, interferon-gamma, and oncostatin M for human melanoma cells. J Immunol. 1987;139: 2977–83. Available: http://www.ncbi.nlm.nih.gov/pubmed/3117884 3117884

[9] Cross A, Edwards SW, Bucknall RC, Moots RJ. Secretion of oncostatin M by neutrophils in rheumatoid arthritis. Arthritis Rheum. Wiley Subscription Services, Inc., A Wiley Company; 2004;50: 1430–1436. 10.1002/art.20166 15146412

[10] Hui W, Bell M, Carroll G. Detection of oncostatin M in synovial fluid from patients with rheumatoid arthritis. Ann Rheum Dis. 1997;56: 184–187. Available: http://ard.bmj.com/content/annrheumdis/56/3/184.full.pdf 9135222 10.1136/ard.56.3.184 PMC1752333

[11] Pradeep AR, Thorat Manojkumar S, Garima G, Raju A. Serum levels of oncostatin M (a gp 130 cytokine): an inflammatory biomarker in periodontal disease. Biomarkers. 2010;15: 277–282. 10.3109/13547500903573209 20408777

[12] West NR, Hegazy AN, Owens BMJ, Bullers SJ, Linggi B, Buonocore S, et al Oncostatin M drives intestinal inflammation and predicts response to tumor necrosis factor–neutralizing therapy in patients with inflammatory bowel disease. Nat Med. 2017;23: 579–589. 10.1038/nm.4307 28368383 PMC5420447

[13] Vasse M, Pourtau J, Trochon V, Muraine M, Vannier J-P, Lu H, et al Oncostatin M Induces Angiogenesis In Vitro and In Vivo. Arterioscler Thromb Vasc Biol. 1999;19 Available: http://atvb.ahajournals.org/content/19/8/1835 10.1161/01.atv.19.8.1835 10446061

[14] Camaré C, Pucelle M, Nègre-Salvayre A, Salvayre R. Angiogenesis in the atherosclerotic plaque. Redox Biol. 2017;12: 18–34. 10.1016/j.redox.2017.01.007 28212521 PMC5312547

[15] Shi N, Chen S-Y. Mechanisms simultaneously regulate smooth muscle proliferation and differentiation. J Biomed Res. Education Department of Jiangsu Province; 2014;28: 40–6. 10.7555/JBR.28.20130130 24474962 PMC3904173

[16] Albasanz-Puig A, Murray J, Preusch M, Coan D, Namekata M, Patel Y, et al Oncostatin M is expressed in atherosclerotic lesions: a role for Oncostatin M in the pathogenesis of atherosclerosis. Atherosclerosis. Elsevier; 2011;216: 292–8. 10.1016/j.atherosclerosis.2011.02.003 21376322

[17] Tenger C, Sundborger A, Jawien J, Zhou X. IL-18 accelerates atherosclerosis accompanied by elevation of IFN-gamma and CXCL16 expression independently of T cells. Arterioscler Thromb Vasc Biol. 2005;25: 791–6. 10.1161/01.ATV.0000153516.02782.65 15604417

[18] Reddy VS, Valente AJ, Delafontaine P, Chandrasekar B. Interleukin-18/WNT1-inducible signaling pathway protein-1 signaling mediates human saphenous vein smooth muscle cell proliferation. J Cell Physiol. 2011;226: 3303–3315. 10.1002/jcp.22676 21321938 PMC3111842

[19] Amin MA, Rabquer BJ, Mansfield PJ, Ruth JH, Marotte H, Haas CS, et al Interleukin 18 induces angiogenesis in vitro and in vivo via Src and Jnk kinases. Ann Rheum Dis. 2010;69: 2204–2212. 10.1136/ard.2009.127241 20679476

[20] Jing Y, Ma N, Fan T, Wang C, Bu X, Jiang G, et al Tumor necrosis factor-alpha promotes tumor growth by inducing vascular endothelial growth factor. Cancer Invest. 2011;29: 485–93. 10.3109/07357907.2011.597812 21740086

[21] Zhang Y, Yang X, Bian F, Wu P, Xing S, Xu G, et al TNF-α promotes early atherosclerosis by increasing transcytosis of LDL across endothelial cells: Crosstalk between NF-κB and PPAR-γ. J Mol Cell Cardiol. 2014;72: 85–94. 10.1016/j.yjmcc.2014.02.012 24594319

[22] Stamatiou R, Paraskeva E, Gourgoulianis K, Molyvdas P-A, Hatziefthimiou A. Cytokines and Growth Factors Promote Airway Smooth Muscle Cell Proliferation. ISRN Inflamm. Hindawi; 2012;2012: 1–13. 10.5402/2012/731472 24049651 PMC3767366

[23] Morel JC, Park CC, Woods JM, Koch AE. A novel role for interleukin-18 in adhesion molecule induction through NF kappa B and phosphatidylinositol (PI) 3-kinase-dependent signal transduction pathways. J Biol Chem. 2001;276: 37069–75. 10.1074/jbc.M103574200 11477102

[24] Makó V, Czúcz J, Weiszhár Z, Herczenik E, Matkó J, Prohászka Z, et al Proinflammatory activation pattern of human umbilical vein endothelial cells induced by IL-1β, TNF-α, and LPS. Cytometry A. 2010;77: 962–70. 10.1002/cyto.a.20952 21290470

[25] Brown TJ, Rowe JM, Liu JW, Shoyab M. Regulation of IL-6 expression by oncostatin M. J Immunol. 1991;147: 2175–80. Available: http://www.ncbi.nlm.nih.gov/pubmed/1918953 1918953

[26] Langenkamp E, Molema G. Microvascular endothelial cell heterogeneity: general concepts and pharmacological consequences for anti-angiogenic therapy of cancer. Cell Tissue Res. Springer-Verlag; 2009;335: 205–222. 10.1007/s00441-008-0642-4 18677515

[27] Aird WC. Endothelial Cell Heterogeneity. Cold Spring Harb Perspect Med. 2012;2: a006429–a006429. 10.1101/cshperspect.a006429 22315715 PMC3253027

[28] Scott DW, Vallejo MO, Patel RP. Heterogenic Endothelial Responses to Inflammation: Role for Differential N-Glycosylation and Vascular Bed of Origin. J Am Heart Assoc. 2013;2: e000263–e000263. 10.1161/JAHA.113.000263 23900214 PMC3828811

[29] Wang Q, Pfeiffer GR, Stevens T, Doerschuk CM. Lung Microvascular and Arterial Endothelial Cells Differ in Their Responses to Intercellular Adhesion Molecule-1 Ligation. Am J Respir Crit Care Med. American Thoracic Society; 2002;166: 872–877. 10.1164/rccm.2201007 12231500

[30] Falk E. Pathogenesis of Atherosclerosis. J Am Coll Cardiol. Elsevier; 2006;47: C7–C12. 10.1016/j.jacc.2005.09.068 16631513

[31] Dewey FE, Gusarova V, Dunbar RL, O’Dushlaine C, Schurmann C, Gottesman O, et al Genetic and Pharmacologic Inactivation of ANGPTL3 and Cardiovascular Disease. N Engl J Med. 2017;377: 211–221. 10.1056/NEJMoa1612790 28538136 PMC5800308

[32] Kühnast S, van der Hoorn JWA, Pieterman EJ, van den Hoek AM, Sasiela WJ, Gusarova V, et al Alirocumab inhibits atherosclerosis, improves the plaque morphology, and enhances the effects of a statin. J Lipid Res. 2014;55: 2103–12. 10.1194/jlr.M051326 25139399 PMC4174003

[33] Sugaya M, Fang L, Cardones AR, Kakinuma T, Jaber SH, Blauvelt A, et al Oncostatin M enhances CCL21 expression by microvascular endothelial cells and increases the efficiency of dendritic cell trafficking to lymph nodes. J Immunol. 2006;177: 7665–72. Available: http://www.ncbi.nlm.nih.gov/pubmed/17114436 17114436 10.4049/jimmunol.177.11.7665

[34] Modur V, Feldhaus MJ, Weyrich AS, Jicha DL, Prescott SM, Zimmerman GA, et al Oncostatin M is a proinflammatory mediator. In vivo effects correlate with endothelial cell expression of inflammatory cytokines and adhesion molecules. J Clin Invest. American Society for Clinical Investigation; 1997;100: 158–68. 10.1172/JCI119508 9202068 PMC508176

[35] Rega G, Kaun C, Demyanets S, Pfaffenberger S, Rychli K, Hohensinner PJ, et al Vascular Endothelial Growth Factor Is Induced by the Inflammatory Cytokines Interleukin-6 and Oncostatin M in Human Adipose Tissue In Vitro and in Murine Adipose Tissue In Vivo. Arterioscler Thromb Vasc Biol. 2007;27: 1587–1595. 10.1161/ATVBAHA.107.143081 17525365

[36] Rychli K, Kaun C, Hohensinner PJ, Rega G, Pfaffenberger S, Vyskocil E, et al The inflammatory mediator oncostatin M induces angiopoietin 2 expression in endothelial cells in vitro and in vivo. J Thromb Haemost. 2010;8: 596–604. 10.1111/j.1538-7836.2010.03741.x 20088942 PMC2857505

[37] Landlinger C, Pouwer MG, Juno C, van der Hoorn JWA, Pieterman EJ, Jukema JW, et al The AT04A vaccine against proprotein convertase subtilisin/kexin type 9 reduces total cholesterol, vascular inflammation, and atherosclerosis in APOE*3Leiden.CETP mice. Eur Heart J. 2017;38: 2499–2507. 10.1093/eurheartj/ehx260 28637178 PMC5837708

[38] Pigott R, Dillon LP, Hemingway IH, Gearing AJ. Soluble forms of E-selectin, ICAM-1 and VCAM-1 are present in the supernatants of cytokine activated cultured endothelial cells. Biochem Biophys Res Commun. 1992;187: 584–9. Available: http://www.ncbi.nlm.nih.gov/pubmed/1382417 1382417 10.1016/0006-291x(92)91234-h

[39] Rawlings JS, Rosler KM, Harrison DA. The JAK/STAT signaling pathway. J Cell Sci. 2004;117: 1281–1283. Available: http://jcs.biologists.org/content/117/8/1281 10.1242/jcs.00963 15020666

[40] Scheller J, Chalaris A, Schmidt-Arras D, Rose-John S. The pro- and anti-inflammatory properties of the cytokine interleukin-6. Biochim Biophys Acta—Mol Cell Res. 2011;1813: 878–888. 10.1016/j.bbamcr.2011.01.034 21296109

[41] Heinrich PC, Behrmann I, Müller-Newen G, Schaper F, Graeve L. Interleukin-6-type cytokine signalling through the gp130/Jak/STAT pathway. Biochem J. 1998;334: 297–314. Available: http://www.ncbi.nlm.nih.gov/pubmed/9716487 9716487 10.1042/bj3340297 PMC1219691

[42] Ruprecht K, Kuhlmann T, Seif F, Hummel V, Kruse N, Brück W, et al Effects of oncostatin M on human cerebral endothelial cells and expression in inflammatory brain lesions. J Neuropathol Exp Neurol. 2001;60: 1087–98. Available: http://www.ncbi.nlm.nih.gov/pubmed/11706938 11706938 10.1093/jnen/60.11.1087

[43] Modur V, Li Y, Zimmerman GA, Prescott SM, McIntyre TM. Retrograde inflammatory signaling from neutrophils to endothelial cells by soluble interleukin-6 receptor alpha. J Clin Invest. 1997;100: 2752–2756. 10.1172/JCI119821 9389739 PMC508479

[44] Kevil CG, Patel RP, Bullard DC. Essential role of ICAM-1 in mediating monocyte adhesion to aortic endothelial cells. Am J Physiol Physiol. 2001;281: C1442–C1447. 10.1152/ajpcell.2001.281.5.C1442 11600406

[45] Tokuhira M, Hosaka S, Volin M V., Haines GK, Katschke KJ, Kim S, et al Soluble vascular cell adhesion molecule 1 mediation of monocyte chemotaxis in rheumatoid arthritis. Arthritis Rheum. John Wiley & Sons, Inc.; 2000;43: 1122 10.1002/1529-0131(200005)43:5<1122::AID-ANR23>3.0.CO;2–7 10817567

[46] Kang S-A, Blache CA, Bajana S, Hasan N, Kamal M, Morita Y, et al The effect of soluble E-selectin on tumor progression and metastasis. BMC Cancer. BioMed Central; 2016;16: 331 10.1186/s12885-016-2366-2 27220365 PMC4879723

[47] Natarelli L, Geißler C, Csaba G, Wei Y, Zhu M, di Francesco A, et al miR-103 promotes endothelial maladaptation by targeting lncWDR59. Nat Commun. Nature Publishing Group; 2018;9: 2645 10.1038/s41467-018-05065-z 29980665 PMC6035258

[48] McDonald AI, Shirali AS, Aragón R, Ma F, Hernandez G, Vaughn DA, et al Endothelial Regeneration of Large Vessels Is a Biphasic Process Driven by Local Cells with Distinct Proliferative Capacities. Cell Stem Cell. 2018;23: 210–225.e6. 10.1016/j.stem.2018.07.011 30075129 PMC6178982

[49] Reynolds HR, Buyon J, Kim M, Rivera TL, Izmirly P, Tunick P, et al Association of plasma soluble E-selectin and adiponectin with carotid plaque in patients with systemic lupus erythematosus. Atherosclerosis. 2010;210: 569–574. 10.1016/j.atherosclerosis.2009.12.007 20044088 PMC3963602

[50] Zhao W, Feng H, Guo S, Han Y, Chen X. Danshenol A inhibits TNF-α-induced expression of intercellular adhesion molecule-1 (ICAM-1) mediated by NOX4 in endothelial cells. Sci Rep. 2017;7: 12953 10.1038/s41598-017-13072-1 29021525 PMC5636799

[51] Dumas A, Lagarde S, Laflamme C, Pouliot M. Oncostatin M decreases interleukin-1 β secretion by human synovial fibroblasts and attenuates an acute inflammatory reaction in vivo. J Cell Mol Med. Wiley-Blackwell; 2012;16: 1274–85. 10.1111/j.1582-4934.2011.01412.x 21854541 PMC3823080

[52] Schnittker D, Kwofie K, Ashkar A, Trigatti B, Richards CD. Oncostatin M and TLR-4 ligand synergize to induce MCP-1, IL-6, and VEGF in human aortic adventitial fibroblasts and smooth muscle cells. Mediators Inflamm. Hindawi; 2013;2013: 317503 10.1155/2013/317503 24307759 PMC3836373

[53] Pugin J, Ulevitch RJ, Tobias PS. A critical role for monocytes and CD14 in endotoxin-induced endothelial cell activation. J Exp Med. 1993;178: 2193–200. Available: http://www.ncbi.nlm.nih.gov/pubmed/7504060 7504060 10.1084/jem.178.6.2193 PMC2191301

[54] Foglia B, Cannito S, Morello E, Turato C, Di Maira G, Novo E, et al Oncostatin M induces increased invasiveness and angiogenesis in hepatic cancer cells through HIF1α-related release of VEGF-A. Dig Liver Dis. Elsevier; 2017;49: e5 10.1016/j.dld.2017.01.013

[55] Fossey SL, Bear MD, Kisseberth WC, Pennell M, London CA. Oncostatin M promotes STAT3 activation, VEGF production, and invasion in osteosarcoma cell lines. BMC Cancer. 2011;11: 125 10.1186/1471-2407-11-125 21481226 PMC3079692

[56] Weiss TW, Simak R, Kaun C, Rega G, Pflüger H, Maurer G, et al Oncostatin M and IL-6 induce u-PA and VEGF in prostate cancer cells and correlate in vivo. Anticancer Res. 2011;31: 3273–8. Available: http://www.ncbi.nlm.nih.gov/pubmed/21965736 21965736

[57] Kim I, Moon S-O, Hoon Kim S, Jin Kim H, Soon Koh Y, Young Koh G. VEGF Stimulates Expression of ICAM-1, VCAM-1 and E-Selectin through Nuclear Factor-κ B Activation in Endothelial Cells Downloaded from [Internet]. JBC Papers in Press; 2000 Available: http://www.jbc.org/

[58] Mudau M, Genis A, Lochner A, Strijdom H. Endothelial dysfunction: the early predictor of atherosclerosis. Cardiovasc J Afr. Clinics Cardive Publishing (Pty) Ltd.; 2012;23: 222–31. 10.5830/CVJA-2011-068 22614668 PMC3721957

[59] Kapoor D, Trikha D, Vijayvergiya R, Kaul D, Dhawan V. Conventional therapies fail to target inflammation and immune imbalance in subjects with stable coronary artery disease: a system-based approach. Atherosclerosis. 2014;237: 623–31. 10.1016/j.atherosclerosis.2014.10.009 25463097

[60] Zhang X, Li J, Qin J-J, Cheng W-L, Zhu X, Gong F-H, et al Oncostatin M receptor β deficiency attenuates atherogenesis by inhibiting JAK2/STAT3 signaling in macrophages. J Lipid Res. 2017;58: 895–906. 10.1194/jlr.M074112 28258089 PMC5408608

[61] Collins RG, Velji R, Guevara N V, Hicks MJ, Chan L, Beaudet AL. P-Selectin or intercellular adhesion molecule (ICAM)-1 deficiency substantially protects against atherosclerosis in apolipoprotein E-deficient mice. J Exp Med. 2000;191: 189–94. Available: http://www.ncbi.nlm.nih.gov/pubmed/10620617 10620617 10.1084/jem.191.1.189 PMC2195808

[62] Gosling J, Slaymaker S, Gu L, Tseng S, Zlot CH, Young SG, et al MCP-1 deficiency reduces susceptibility to atherosclerosis in mice that overexpress human apolipoprotein B. J Clin Invest. American Society for Clinical Investigation; 1999;103: 773–8. 10.1172/JCI5624 10079097 PMC408147

[63] Esashi E, Ito H, Minehata K, Saito S, Morikawa Y, Miyajima A. Oncostatin M deficiency leads to thymic hypoplasia, accumulation of apoptotic thymocytes and glomerulonephritis. Eur J Immunol. WILEY‐VCH Verlag; 2009;39: 1664–1670. 10.1002/eji.200839149 19384873

[64] Kisucka J, Chauhan AK, Patten IS, Yesilaltay A, Neumann C, Van Etten RA, et al Peroxiredoxin1 Prevents Excessive Endothelial Activation and Early Atherosclerosis. Circ Res. 2008;103: 598–605. Available: http://circres.ahajournals.org/content/103/6/598 10.1161/CIRCRESAHA.108.174870 18689572 PMC4911701

[65] Rajashekhar G, Willuweit A, Patterson CE, Sun P, Hilbig A, Breier G, et al Continuous endothelial cell activation increases angiogenesis: evidence for the direct role of endothelium linking angiogenesis and inflammation. J Vasc Res. Karger Publishers; 2006;43: 193–204. 10.1159/000090949 16410682

[66] Wilder RL, Case JP, Crofford LJ, Kumkumian GK, Lafyatis R, Remmers EF, et al Endothelial cells and the pathogenesis of rheumatoid arthritis in humans and streptococcal cell wall arthritis in Lewis rats. J Cell Biochem. 1991;45: 162–166. 10.1002/jcb.240450207 2055944

[67] Zwaginga JJ, Sixma JJ, de Groot PG. Activation of endothelial cells induces platelet thrombus formation on their matrix. Studies of new in vitro thrombosis model with low molecular weight heparin as anticoagulant. Arteriosclerosis. 10: 49–61. Available: http://www.ncbi.nlm.nih.gov/pubmed/2297347 2297347 10.1161/01.atv.10.1.49

