# Supplementary figures and images for: Inflammatory cytokine oncostatin M induces endothelial activation in macro- and microvascular endothelial cells and in APOE*3Leiden.CETP mice
Source: PLoS One. 2018 Oct 1;13(10):e0204911. doi: 10.1371/journal.pone.0204911 (PMC6166945; doi:10.1371/journal.pone.0204911)

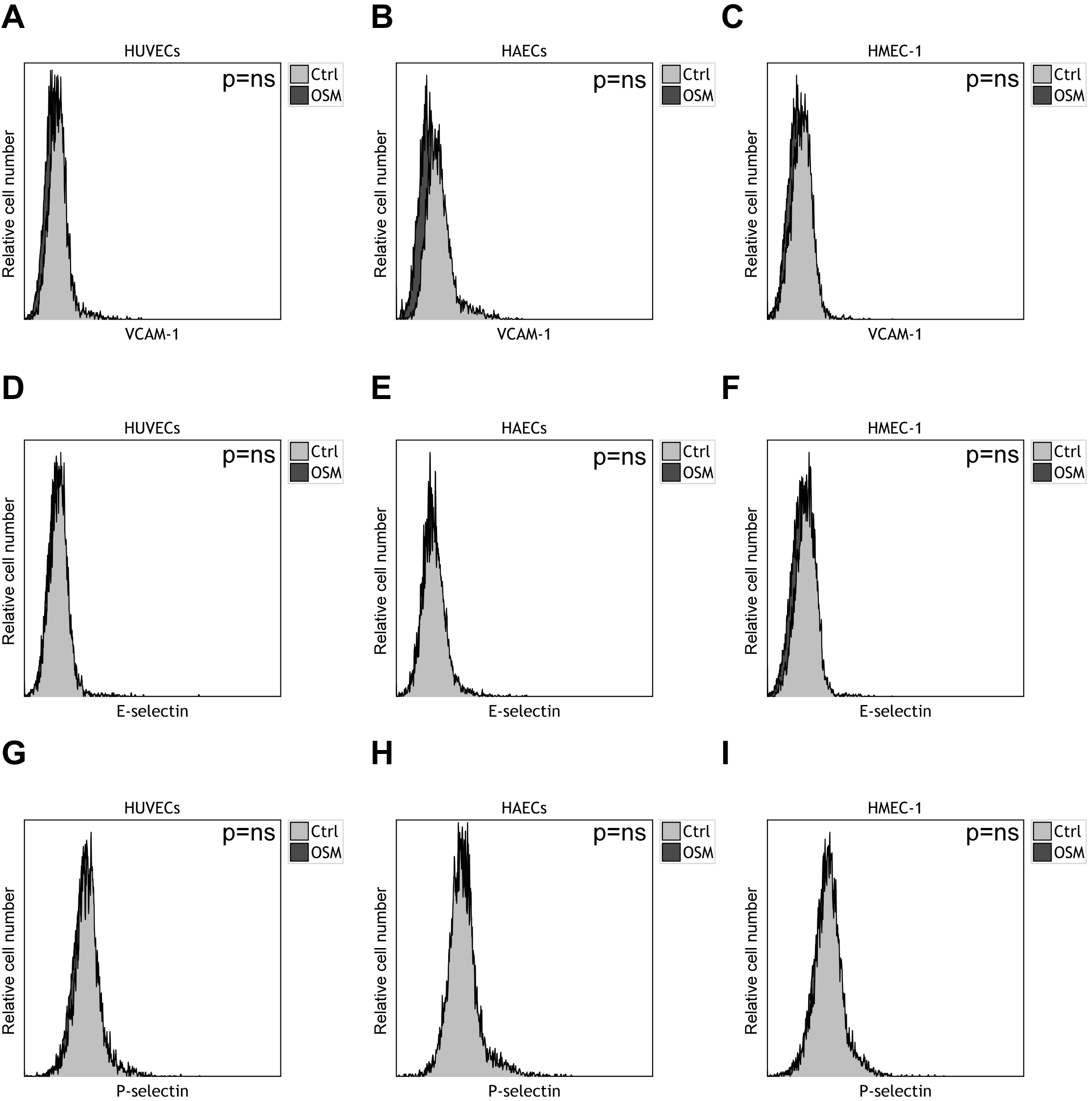

Supplement: S1 Fig — HUVECs, HAECs and HMEC-1 cells were incubated with 5 ng/mL OSM for 18h. A two-way ANOVA with Dunnett’s test was performed on the median to test for significance (n = 3). ns = not significant. (TIF) [file pone.0204911.s001.tif]
