# Supplementary material for: Inflammatory cytokine oncostatin M induces endothelial activation in macro- and microvascular endothelial cells and in APOE*3Leiden.CETP mice
Source: PLoS One. 2018 Oct 1;13(10):e0204911. doi: 10.1371/journal.pone.0204911 (PMC6166945; doi:10.1371/journal.pone.0204911)
Supplement: S1 Table — (PDF) [file pone.0204911.s002.pdf]

Primary antibodies	Clone	Reactivity	Species/Isotype	Dilution	Company	Cat. No
**VCAM-1 conjugated with FITC**	STA	Human	Mouse IgG	2.5 µL/ 1,000,000 cells	Thermo Fisher, Waltham, MA	11-1069-42
**ICAM-1 conjugated with PerCP-eFluor 710**	HA58	Human	Mouse IgG1	2.5 µL/ 1,000,000 cells	Thermo Fisher, Waltham, MA	46-0549-42
**P-selectin conjugated with APC**	Psel.KO2.3	Human, Mouse	Mouse IgG	2.5 µL/ 1,000,000 cells	Thermo Fisher, Waltham, MA	17-0626-82
**E-selectin conjugated with PE**	P2H3	Human	Mouse IgG	2.5 µL/ 1,000,000 cells	Thermo Fisher, Waltham, MA	12-0627-42
**ICAM-1**		Human Rat mouse	Mouse monoclonal IgG_{2a}	1:400	Santa Cruz Biotechnology, Dallas, TX	sc-8439
**AIA 31240**		Mouse	Rabbit	1:500	Accurate Chemical and Scientific, Westbury, NY	J1857
**STAT1**		Human, Mouse, Rat, Monkey	Rabbit	1:1,000	Cell signaling, Danvers, MA	9172
**phosphoSTAT1 (Tyr701)**	58D6	Human, Mouse	Rabbit IgG	1:1,000	Cell signaling, Danvers, MA	9167
**STAT3**	D3Z2G	Human, Mouse, Rat, Monkey	Rabbit IgG	1:1,000	Cell signaling, Danvers, MA	12640
**phosphoSTAT3 (Tyr705)**	D3A7	Human, Mouse, Rat, Monkey	Rabbit IgG	1:2,000	Cell signaling, Danvers, MA	9145
**GAPDH**	14C10	Human, Mouse, Rat, Monkey, Bovine, Pig	Rabbit	1:7,500	Cell signaling, Danvers, MA	2118S

Secondary antibodies	Reactivity	Isotype	Dilution	Company	Cat. No
**Goat Anti-Rabbit antibody conjugated with HRP**	Rabbit	Goat	1:2,000	Dako, Glostrup, Denmark	P0448
**Goat Anti-Mouse antibody conjugated with HRP**	Mouse	Goat	1:2,000	Dako, Glostrup, Denmark	P0447
