# Supplementary material for: Inflammatory cytokine oncostatin M induces endothelial activation in macro- and microvascular endothelial cells and in APOE*3Leiden.CETP mice
Source: PLoS One. 2018 Oct 1;13(10):e0204911. doi: 10.1371/journal.pone.0204911 (PMC6166945; doi:10.1371/journal.pone.0204911)
Supplement: S2 Table — (PDF) [file pone.0204911.s003.pdf]

	**Dose** (µg/kg/day)	**Food intake** (g/mouse/day)	**ALT** (U/L)	**AST** (U/L)
**Control**	-	2.4 ± 0.2	53.1	324
**OSM**	1	2.7 ± 0.4	52.5	333
**OSM**	3	2.4 ± 0.3	92.1	669
**OSM**	10	2.4 ± 0.3	59.4	293
